# Functional Recovery Associated with Dendrite Regeneration in PVD Neuron of *Caenorhabditis elegans*

**DOI:** 10.1523/ENEURO.0292-23.2024

**Published:** 2024-05-15

**Authors:** Harjot Kaur Brar, Swagata Dey, Pallavi Singh, Devashish Pande, Anindya Ghosh-Roy

**Affiliations:** Department of Cellular and Molecular Neuroscience, National Brain Research Centre, Manesar 122052, Haryana, India

**Keywords:** *Caenorhabditis elegans*, dendrite regeneration, dendrotomy, harsh touch, posture, PVD neuron

## Abstract

PVD neuron of *Caenorhabditis elegans* is a highly polarized cell with well-defined axonal, and dendritic compartments. PVD neuron operates in multiple sensory modalities including the control of both nociceptive touch sensation and body posture. Although both the axon and dendrites of this neuron show a regeneration response following laser-assisted injury, it is rather unclear how the behavior associated with this neuron is affected by the loss of these structures. It is also unclear whether neurite regrowth would lead to functional restoration in these neurons. Upon axotomy, using a femtosecond laser, we saw that harsh touch response was specifically affected leaving the body posture unperturbed. Subsequently, recovery in the touch response is highly correlated to the axon regrowth, which was dependent on DLK-1/MLK-1 MAP Kinase. Dendrotomy of both major and minor primary dendrites affected the wavelength and amplitude of sinusoidal movement without any apparent effect on harsh touch response. We further correlated the recovery in posture behavior to the type of dendrite regeneration events. We found that dendrite regeneration through the fusion and reconnection between the proximal and distal branches of the injured dendrite corresponded to improved recovery in posture. Our data revealed that the axons and dendrites of PVD neurons regulate the nociception and proprioception in worms, respectively. It also revealed that dendrite and axon regeneration lead to the restoration of these differential sensory modalities.

## Visual Abstract

**Figure:**
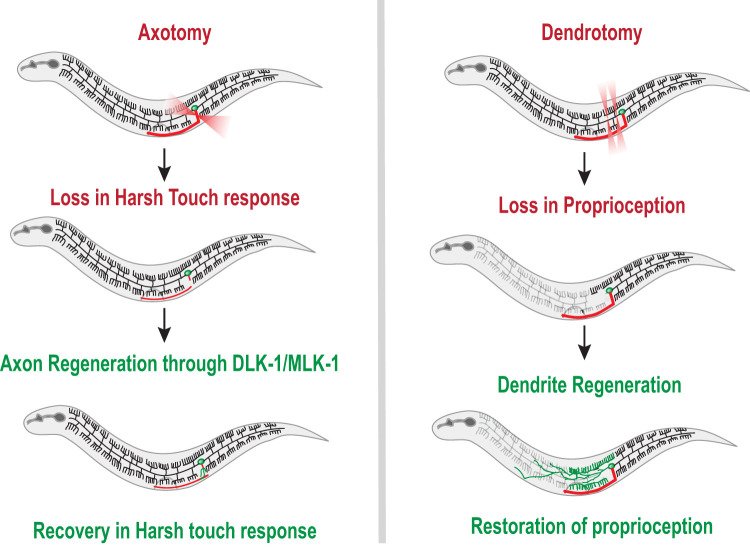

Differential regulation of sensory modalities by the axon and dendrites of PVD neuron. The visual abstract summarizes the roles of the axonal and dendritic compartments in the nociceptive touch response and proprioception in worms, respectively. Loss of axon and dendrite cause a decline in harsh touch and posture, respectively which is regained when these compartments regenerate.

## Significance Statement

Nervous system injury can lead to a wide range of functional impairments including loss of sensations and paralysis. It is often seen that regrowth of the injured axon leads to functional recovery. As nervous system injury is not just limited to axons, the regenerative capacity of injured dendrites also begs our attention. To address this, we severed both the axon and dendrites of PVD neurons in *Caenorhabditis elegans* using laser. We found that axotomy and dendrotomy lead to exclusive loss of touch sensation and proprioception, respectively. Subsequently, we noticed that rewiring of the injured axon leads to recovery in touch response. Whereas dendrite regeneration leads to improvement in proprioception, this work highlights the importance of dendrite repair after physical injury.

## Introduction

Both axons and dendrites are vulnerable to damage upon accidental injury (Park et al., 1996; Mizielinska et al., 2009; Risher et al., 2010). The molecular mechanism of axon regeneration has been highly studied over the last few decades as the regrowth from the injured axonal stump and its rewiring leads to functional recovery (Laha et al., 2017; Rasmussen and Sagasti, 2017; Basu et al., 2021; Winter et al., 2022; Zheng and Tuszynski, 2023). The manipulation of the cAMP pathway, mTOR signaling, and epigenetic factors led to functional recovery following the injuries to central nervous system in vertebrate models (Qiu et al., 2002; Bei et al., 2016; Weng et al., 2017). The precise injury to axons using ultrafast lasers in models such as nematode, fruitfly, and zebrafish opened the window to understanding the mechanism of axon regeneration (Yanik et al., 2004; Ghosh-Roy and Chisholm, 2010; He and Jin, 2016; Richardson and Shen, 2019). A p38 MAP kinase cascade involving Dual leucine zipper kinase kinase kinase (DLK-1) is essential for initiating axon regeneration from the cut tip of the axon in various models (Hammarlund et al., 2009; Yan et al., 2009; Ghosh-Roy et al., 2010; Shin et al., 2012). Manipulation of *let-7* miRNA, Insulin (IIS) signaling, and AMP kinase cascade can help overcome the age-related decline in axon regeneration in *Caenorhabditis elegans* (Zou et al., 2013; Basu et al., 2017; Wang et al., 2018; Kumar et al., 2021). In comparison to axon regeneration, the regeneration response following dendrite injury is less studied (Thompson-Peer et al., 2016; Oren-Suissa et al., 2017; Stone et al., 2022).

The highly branched dendrites of *Drosophila* da sensory neurons and dendrites of PVD neurons in *C. elegans* can regrow following injury (Thompson-Peer et al., 2016; Oren-Suissa et al., 2017; Brar et al., 2022). Both in flies and worms, dendrite regeneration is independent of the conserved DLK-1 MAP Kinase pathway (Stone et al., 2014; Brar et al., 2022), which is essential for axon regeneration (He and Jin, 2016). The dendrites of motor neurons can regenerate in the zebrafish and mice spinal cord (Stone et al., 2022; Li et al., 2023). Therefore, understanding the molecular mechanism regulating dendrite regeneration and its functional significance are endeavors of high importance. After the dendrotomy of class IV da neuron in fly, the affected nociceptive function is recovered through dendrite regrowth (Thompson-Peer et al., 2016; Hertzler et al., 2023). However, these experiments are done in larval stages. Therefore, regrowth seen in fly dendrites after a laser injury could partly be overlapping with the remodeling process during larval development (Yaniv and Schuldiner, 2016).

PVD neurons in *C. elegans* are recognized for a range of behavioral responses to external stimuli, including harsh touch response, thermo-sensation, sound sensing, and proprioception (Chatzigeorgiou et al., 2010; Albeg et al., 2011; Ibsen et al., 2015; Tao et al., 2019; Iliff et al., 2021). The DEG/ENaC channel TRP-4 and ASIC-1 in PVD is responsible for detecting the harsh touch (Li et al., 2011, Husson et al., 2012). PVD neuron is also responsible for the optimal values of the amplitude and wavelength of the sinusoidal traces made by a moving worm on a bacterial lawn (Albeg et al., 2011; Tao et al., 2019). A recent study shows that the PVD dendrites generate local calcium influx and release neuropeptide NLP-12 neuropeptide during locomotion, which directly modulates neuromuscular junction activity (Tao et al., 2019). This local calcium influx is dependent on the DEG/ENaC channels MEC-10, UNC-8, and DEL-1 (Tao et al., 2019). The same study indicated that the DEG/ENaC/ASIC channel, DEGT-1 acts as a mechanoreceptor for harsh touch, which is transduced through the axon to the downstream interneuron (Tao et al., 2019). Therefore, the dendrites and axons of PVD neurons could differentially regulate the proprioception and harsh touch sensation behaviors independently.

In this work, we analyzed both harsh touch sensation and body posture after the axotomy or dendrotomy using a femtosecond laser. We discovered that injury to primary dendrites exclusively affects the body posture while leaving the harsh touch response unchanged, whereas the axotomy affects harsh touch sensitivity without altering the proprioception. At later time points, the animals suffered from axotomy-induced loss of touch sensation regained their function in a DLK-1/MLK-1 MAP kinase-dependent manner. The behavioral recovery related to proprioception after dendrotomy depends on the pattern of the regeneration response. The recovery in body posture parameters is highly correlated with the fusion events between the proximal and distal dendritic branches of the injured PVD. Our data highlight the functional significance of dendrite regeneration in adulthood.

## Materials and Methods

### Strains of *C. elegans*

*C. elegans* strains were grown and maintained at 20°C on the nematode growth medium (NGM) plates seeded with the OP50 bacteria (Brenner, 1974). The loss of function mutants is represented as (0), for example, the *mlk-1* loss-of-function allele *ok2471* is mentioned as *mlk-1(0)*. Unless otherwise specified, the mutants used in this study are deletion or substitution mutants. The strains used in this study are listed in Extended Data Table 1-1. The Caenorhabditis Genetics Centre (CGC) provided these strains, which were genotyped using the appropriate genotyping primers. The wild type background that was used in most of the experiments is *wdIs52* which expresses diffusible GFP under the PVD neuron specific promoter of gene *F49H12.4* (Smith et al., 2010). An extrachromosomal transgene *shrEx472* that expresses the plasmid pNBRGWY124 (*Pser2prom4[4.1kb]::mScarlet*) was used to confirm the behavioral data obtained in the *wdIs52* background.

10.1523/ENEURO.0292-23.2024.t1-1 Table 1-1 Accounts for the list of *C. elegans* strains used in this study Download Table 1-1, XLSX file.

### Dendrotomy and axotomy

Dendrotomy and axotomy experiments were performed on *C. elegans* using a Bruker system equipped with a SpectraPhysics femtosecond multiphoton laser system and an Olympus 60×/0.9NA water objective. The neurite imaging and severing were performed using 920 and 720 nm wavelengths, respectively. Worms were mounted with the cover glass on 5% agarose pads with a drop of polystyrene beads (Polysciences 00876-15) as a friction-raising agent. In some experiments, Levamisole hydrochloride (10 mM) (Sigma L0380000) was used as a paralyzing pharmacological agent (catalog #2855-25).

**Figure 1.**
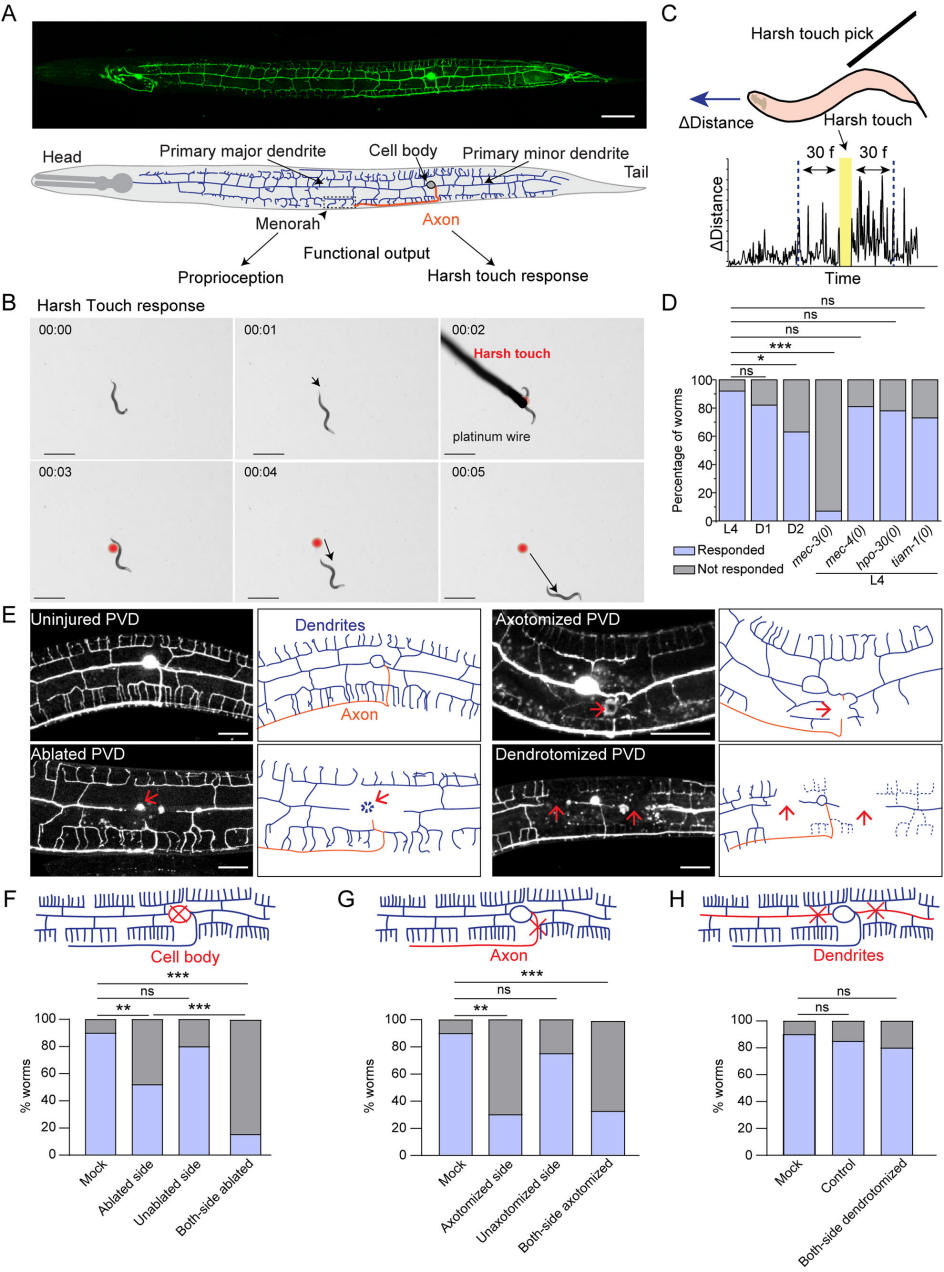
Nociceptive response to harsh touch depends on the integrity of PVD axons but not the dendrites. ***A***, Confocal image of PVD neuron expressing *wdIs52* (*pF49H12.4:: GFP*) reporter and the schematics showing dendrites in blue, and axon in orange color. The scale bar represents the 20 microns value. ***B***, Time-lapse video montage showing worm's response before and after the harsh touch by platinum wire. The worm is placed on the NGM plate. The black arrow represents the worm's distance covered in each frame. The red dot indicates the location of the worm where the harsh touch was made. The scale bar is 1 mm. Time-frames are indicated in min:s.as also shown in Movie 1. ***C***, Illustration of “harsh touch response” experiment and an example of time versus instantaneous distance travelled before and after touch stimulus. The distance-values from 30 frames duration, before and after the delivery of touch stimulus were used to calculate the speed of worm and harsh touch response index (HTRI) as shown in Extended Data Fig. 1-1. ***D***, The percentage of worms responding to the harsh touch is shown for L4, Day 1 adult, and Day 2 adult worms along with *mec-3(0)*, *mec-4(0)*, *hpo-30(0)*, and *tiam-1(0)* at L4 stage. The related HTRI values are presented in Extended Data Fig. 1-1C. 15 < *n* < 25, *N* = 3. ***E***, The confocal images of uncut, ablated, axotomized, and dendrotomized PVD neuron at 3–6 h after injury are shown along with the illustrations for dendrites and axon. The red arrow marks the site of injury. The scale bar represents 20 microns. ***F,G***, Percentage of L4 *wdIs52* transgenic worms responding to harsh touch with one or both PVD neurons ablated (***F***) or axotomized (***G***) compared to mock injured worms. For one-sided injured worms, harsh touch was given on both injured (ablated or axotomized) and uninjured (unablated or nonaxotomy) sides. The data is plotted in a population-based contingency plot. 15 < *n* < 25, *N* = 3. The type of injury is shown in schematics with the red cross mark. ***H***, Percentage of L4 *wdIs52* worms responding to harsh touch in control (uninjured), mock, and both-side (PVDL and PVDR) dendrotomy injury conditions. 12 < *n* < 28, *N* = 3. The type of injury is shown in schematics with the red cross mark. The data is plotted in a population-based contingency plot. The statistical analysis for ***D,F–H***, was Fisher's two-tailed exact test with *p*-value as *p* < 0.05*, 0.01**, and 0.001***. ns, not significant; *N*, independent replicates; and *n*, number of worms taken for behavioral study.

**Figure 2.**
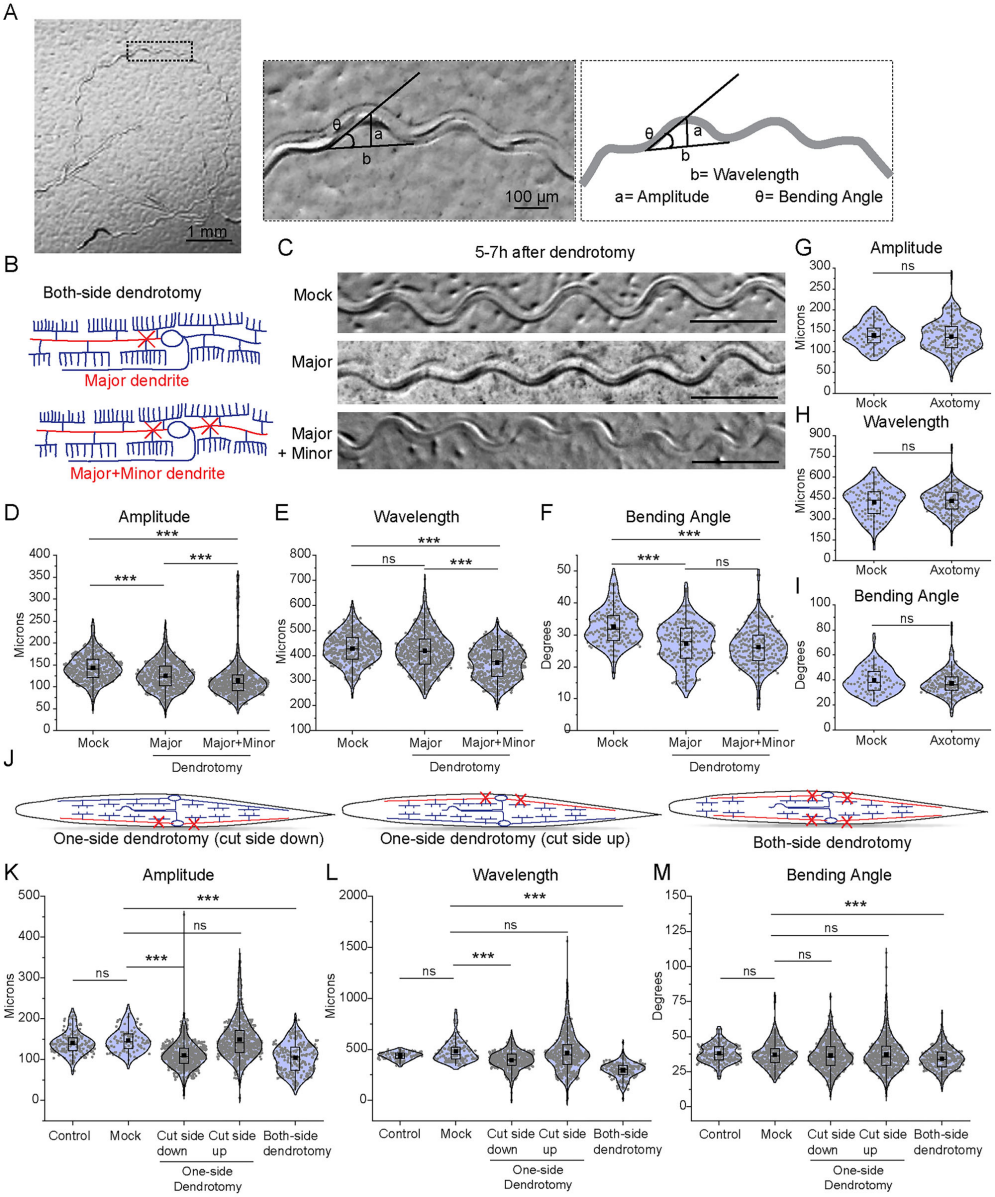
Dendrite injury in PVD results in a defective posture of the worms. ***A***, The worm's trajectory is shown into the OP50 bacterial lawn after a movement for 3–5 min duration. The magnified picture and schematics displaying the postural parameters such as amplitude (a), wavelength (b), and bending angle (θ) are shown. These parameters were also measured at different stages of the worm (Extended Data Fig. 2-1G–I) as well as in different mutant backgrounds Extended Data (Fig. 2-1A–C). ***B***, Schematics representing the cutting experiments including the dendrotomy of “major dendrite only” and dendrotomy of both “major and minor primary dendrites” of both of the PVD neurons (PVDL and PVDR) of the worm are shown. ***C***, Trajectories at 5–7 h after dendrotomy in mock, dendrotomy in the major dendrite (both PVDs), and dendrotomy in the major and minor dendrite (both PVDs) in *wdIS52 (pF49H12.4::GFP)* background worms are shown. The scale bar represents 500 microns. ***D–F***, Posture characteristics such as amplitude (***D***), wavelength (***E***), and bending angle (***F***) in *wdIs52 (pF49H12.4::GFP)* worms are plotted in mock and dendrotomy paradigms mentioned in ***B,C***, 25 < *n* < 32, *N* = 3. The absolute data were displayed, and the violin plots represented the median (red line) and population distribution. The baseline speed and gentle touch response are also plotted (Extended Data Fig. 2-1K,L). ***G–I***, Posture characteristics such as amplitude (***G***), wavelength (***H***), and bending angle (***I***) in *wdIs52 (pF49H12.4::GFP)* worms are plotted in mock, axotomy and ablation (both PVDs) conditions done at Day 1 old worms, 13 < *n* < 32, *N* > 3. The absolute data were displayed, and the violin plots represented the median (red line) and population distribution. ***J***, Schematics of one-side dendrotomy (PVDL or PVDR) and both-side dendrotomy injury where both major and minor dendrites are injured. ***K–M***, The quantification of posture parameters, i.e., amplitude (***K***), wavelength (***L***), and bending angle (***M***) were measured and plotted for control, mock, one-side dendrotomy (cut-side down, i.e., the injured PVD neuron is touching the agar media and cut-side up, i.e., injured PVD neuron is not touching the agar media), and both-side dendrotomy at 8 h after injury. The absolute values are plotted with a red dotted line representing the median of the population data. 27 < *n* < 30, *N* = 3. The statistical analysis for ***D–F,K–M*** is one-way ANOVA with Tukey's multiple comparisons, ***G–I***, student *t* test, with *p* < 0.05*, 0.01**, and 0.001***, ns, not significant. *N* stands for the number of independent replicates, and *n* stands for the number of events.

Usually for dendrotomy, two laser shots were delivered to create a big gap of 10 μm in the primary dendrites (Fig. 1*E*) of PVD as described before (Brar et al., 2022). The first laser shot was delivered at the junction of the first secondary (9–10 µm from the cell body), and the second at a 10 μm distance from the first cut site. The axon of the PVD neuron was severed at 5 μm away from the cell body, resulting in a noticeable gap (Fig. 1*E*). As a control, mock injuries were carried out away from the PVD neurites with an equivalent number of laser shots to other cohorts of injuries performed in any particular experiment. The injured worms were then recovered to OP50-seeded NGM plates using a mouth pipette. To assess the behavior of the injured PVD, injury paradigms of ablation and axotomy involving either one PVD (PVDL or PVDR) or both (PVDL and PVDR) are mentioned in the respective figure panels. For dendrotomy, one paradigm involved injuring major and minor dendrites of either one PVD neuron (PVDL or PVDR) or both the PVDs (Fig. 2*J*). Second dendrotomy paradigm involved injuring either the major dendrite or major and minor dendrites of both the PVD neurons to understand the role of the major dendrite specifically (Fig. 2*B*).

10.1523/ENEURO.0292-23.2024.f1-1 Fig 1-1 Harsh touch response index measured in different injury conditions of the PVD neurons (A) The formula of speed and Harsh touch response index (HTRI) is shown which was used in the analysis of injury experiments. The speed of the worm was measured considering 30 frames before and after harsh touch which was labeled as speed before harsh touch and speed after harsh touch, respectively. The ratio of these speeds was taken as HTRI. (B) The values of speed of uninjured worms are plotted before and after harsh touch at L4, Day 1, and Day 2 old stage worms on the NGM plates are shown in microns per second. 12 < n < 30, N = 3. (C) Harsh touch response indices are plotted for L4, Day1, Day2, L4 (*mec-3(0)*) and L4 (*mec-4(0)*) worms in *wdIs52 (pF49H12.4::GFP)* background. The violin plots represent the median (red line) and population distribution. 12 < n < 25, N = 3. (D-F) Harsh touch response indices are plotted for ablation (D), axotomy (E), and dendrotomy (F) experiments. Each violin plot in (D) represents mock, one side ablation (ablated-side, and unablated side), and both side ablation in *wdIs52 (pF49H12.4::GFP)* background. 15 < n < 25, N = 3. (E) plot represents mock, one side axotomy (axotomized-side, and non-axotomy side) and both side axotomy in *wdIs52 (pF49H12.4::GFP)* background. 13 < n < 35, N = 3 and (F) represents control (uncut), mock, and both side dendrotomy in *wdIs52 (pF49H12.4::GFP)* background. 12 < n < 28, N = 3. The violin plots represent the median (red line) and population distribution. The statistical analysis for (B-F), is one-way ANOVA with Tukey’s multiple comparisons with p-value as p < 0.05*, 0.01**, and 0.001***. ns stands for not significant, N stands for the number of independent replicates, and n stands for the number of worms taken for behavioural study. Download Fig 1-1, TIF file.

10.1523/ENEURO.0292-23.2024.f2-1 Fig 2-1 **The posture function of PVD neurons is correlated to its dendritic structure.** (A-C) Posture parameters of uninjured worms i.e. amplitude(A), wavelength (B), and bending angle (C) are plotted in *wdIs52, hpo-30(0);wdIs52, mec-3(0);wdIs52, mec-4(0);wdIs52, tiam-1(0) ;wdIs52, and mec-10(0)* at L4 stage are plotted, 25 < n < 30, N = 3. The absolute values were plotted and the violin plots represent the median and population distribution. (D-F) Posture parameters of mock and PVD ablated worms i.e. amplitude(D), wavelength (E), and bending angle (F) are plotted in *wdIs52,* at L4 stage are plotted, 12 < n < 14, N = 3 . (G-I) Posture parameters such as amplitude (G), wavelength (H), and bending angle (I) are plotted in L4, Day1, and Day2 worms respectively. The absolute values were plotted and violin plots represent the median (red line) and population distribution. 15 < n < 25, N > 3. (J) The schematics showing the type of injury performed on *pserprom3::mscarlet* worms using a 2-Photon laser. The PVD neuron is labeled in red and the black cross represents the site of injury. (K-L) The baseline speed (K), as well as gentle touch response (L), were measured for the worms that had undergone dendrotomy of major dendrite (both PVDs) and dendrotomy of major and minor dendrites (both PVDs) of *pser2prom3::mscarlet* worms, 13 < n < 25, N > 3. (M) The images of trajectories at 5-7  h after dendrotomy at Day 1 stage *pser2prom3::mscarlet* worms in mock, Dendrotomy in the major dendrite (both PVDs) and dendrotomy in the major and minor dendrite (both PVDs) are shown. (N-P) Posture parameters i.e. amplitude (N), wavelength (O), and bending angle (P) are plotted in mock, dendrotomy in the major dendrite (both PVDs) and dendrotomy in the major and minor dendrite (both PVDs) in Day1 *pser2prom3::mscarlet* worms. 15 < n < 20, N = 2. The absolute values were plotted and the violin plots represent the median and population distribution. The statistical analysis for A-G, J-L is one-way ANOVA with Tukey’s multiple comparisons, and for (G-bottom panel) is –Fisher's two-tailed exact test with p < 0.05*, 0.01**, and 0.001***. The violin plots represent the population distribution. ns stands for not significant, N stands for the number of independent replicates, and n stands for the number of worms. Download Fig 2-1, TIF file.

The worms that were selected for studying their behavior were observed at 7–8 and 24 h following the injury. The initial sets of injuries were imaged and verified to be accurate with around 87% confirmation rate. The images that are shown in the figures are for the sake of representation.

### Imaging

To measure the degree of regeneration, injured worms were imaged at 24 h after the injury. The worms were immobilized and plated on 5% agarose (Sigma) pads in 10 mM Levamisole hydrochloride (Sigma) media. The worms were scanned using a Nikon A1R confocal system and a tile imaging module with a 60×/1.4NA oil objective to measure the degree of regeneration.

### Dendrite and axon regeneration analysis and quantification

The proximal and distal ends of injured PVD dendrites can fuse after injury (Kravtsov et al., 2017). The overlaps between the regenerating proximal primary dendrite with distal primary dendrite (Fig. 4*F*, green arrowhead) or distal menorah (Fig. 4*F*, green arrowhead) were classified as reconnection phenomena as also described before (Brar et al., 2022). The menorahs having more than one secondary dendrite (Fig. 4*F*, faint red rectangular boxes) were considered as menorah–menorah fusion and the percentage of PVD neurons showing menorah–menorah fusion was calculated and plotted.

Analysis of the axon regeneration was carried out by visual observation and quantitative information was obtained using various image analysis modules of ImageJ. Neurites were traced and their lengths were quantified using the Simple Neurite Tracer plugin of ImageJ (Schindelin et al., 2012). Neurite development from the axon's split point was classed as regenerative growth including the ectopic branches that originate from the cell body, the nearby dendrite, and the overall branch length. To collect statistical data, ImageJ quantifications were further examined in Excel and Graphpad.

### Behavioral analysis of harsh touch response

In this assay, we used a 0.15 mm thick platinum wire in a cuboid form, which has previously been used to provide a harsh touch of 100–200 N force (Li et al., 2011). The worms were allowed to roam freely on the NGM medium plates and harsh touch was provided near the vulva. The assay involved providing the harsh touch by the platinum wire on both left and right sides of the worm, categorized separately for either side and later correlated for the injured (ablated/axotomized/dendrotomized cut side down) and uninjured (unablated/nonaxotomy/cut side up) side in cases of one PVD injured (PVDL or PVDR). The worms displayed an escape response with increased velocity or changed direction or both. The live imaging of the touch response was done using a Leica stereoscope (LEICA M165 FCM165FC) with a camera (LEICA MC120 HD) attached. The recorded videos were analyzed using the worm tracker plugin in the ImageJ Fiji program (Nussbaum-Krammer et al., 2015). The proportion of worms displaying an escape response after the harsh touch to the total number of worms assessed was quantified as the percentage of worms “responded”. Similarly, the worms without any escape response following harsh touch have been classified as a percentage of worms “not responded.”

To quantify the response to the harsh touch, each of the worms was recorded for 30 s before and after the harsh touch. Each worm was assigned under a batch and the data that is obtained is also named under the batch – wise nomenclature. The quantitative strategy was carried out by utilizing the ImageJ plugin “wrmtrck” (Nussbaum-Krammer et al., 2015). For each worm, before and after the harsh touch videos were segregated and analyzed. After transforming the videos into 8-bit videos and thresholded, the wrmtrck plugin was used with parameters such as (minsize = 1,000, maxsize = 19,999, maxvelocity = 800, maxareachange = 900, mintracklength = 2, bendthreshold = 1, binsize = 0, showpathlengths, showlabels, showpositions, smoothingrawdata = 2, benddetect = 0, fps = 10, backsub = 0, threshmode = Otsu, fontsize = 16). The average speed values were calculated from the videos of 30-second duration before and after harsh touch. The 30 frames before the harsh touch were used to calculate speed before the harsh touch and 30 frames after the harsh touch were used to calculate speed after the harsh touch. The ratio of speeds after the harsh touch to before the harsh touch was termed a harsh touch response index or HTRI (Extended Data Fig. 1-1A).

### Behavioral analysis of the posture of worm

The worms were put onto the 3 d old seeded OP50 lawn using an eyelash pick and were let to freely crawl for 3–5 min. The proprioception assay involved placing the worm with their left and right sides on the OP50 lawn, categorized separately for either side, and later correlated for the injured and uninjured side in cases of one PVD injured (PVDL or PVDR). For the posture assay, still images of the sinusoidal trajectories on OP50 lawn were acquired with a Leica camera (LEICA MC120 HD) after the worms were allowed to move for 5 min. The posture parameters included analysis of the amplitude, wavelength, and bending angle of each sinusoidal wave manually determined using Fiji ImageJ software (Fig. 2*A*). The amplitude (a) was measured using a line segment measuring the height of the peak (Fig. 2*A*, “a”). The wavelength (b) was measured using a line segment that connects the two minima (Fig. 2*A*, “b”). The bending angle (θ) was measured using the angle tool of Image J software measuring the angle of the trajectory from the normal (Fig. 2*A*, “θ”). Approximately 30–60 waves per trajectory were taken into account for a single worm. The dataset to be utilized for the statistical analysis includes pooled values of all worms in a certain group. To straighten the traces for representation, the Straighten tool of ImageJ is used based on a segmented line with a line width of 50 spanning the entire width of the trajectory (Fig. 2*A*).

### Gentle touch assay of worm

The freely moving worms were delivered with gentle touch with an eyelash alternatively to the anterior and posterior side as described before. 10 consecutive touches were given to the worm and touch response were presented as posterior touch response index (PTRI) and anterior touch response index (ATRI).

### Double-blinded analysis of worm behavior

We double-blinded the behavioral assays in this study by involving two researchers for a behavior experiment after dendrotomy or axotomy. The worms were injured using two-photon lasers by one researcher and pseudo-labeled. The other researcher then carried out the behavioral assay and analysis on these pseudo-labeled worms. For example, dendrotomy experiments were done in three groups, mock, dendrotomy in major, and both minor plus major of one side of PVD neuron by one researcher and each cohort was pseudo-labeled. Later behavioral studies and regeneration imaging were performed on these worms by another researcher, unaware of the conditions of these cohorts. Each of these cohorts was then assessed as per the categorization of pseudo labels. Behavior such as proprioception and harsh touch was done on these worms at two time points, i.e., 8 and 24 h. The behavioral observations were made for both the left and right sides of the worm and categorized separately. Later on, the regeneration pattern at 24 h was imaged for the same worms using a confocal microscope. The information on behavioral and regeneration patterns was saved for each worm and then correlated for further studies. Based on the side of the worm receiving the harsh touch or placed on the substratum, behavioral readouts were classified for the injured (ablated/axotomized/cut side down) and uninjured PVD (unablated/nonaxotomy/cut side up) in cases of only one PVD injured (PVDL or PVDR).

### Statistical analysis

To prepare statistical analyses, the GraphPad Prism application (Prism 8 V8.2.1) was utilized. Two samples were evaluated using the unpaired two-tailed *t* test. To do statistical analysis on a large number of samples, one-way ANOVA with Tukey's multiple comparisons test was carried out. By using a two-tailed chi-square test to compare population data, Fisher's exact contingency test was employed to compare fraction values for each sample. For each plot, the legends show the number of samples (*n*) and biological replicates (*N*). Significance values of *p* < 0.05*, 0.01**, and 0.001*** obtained through statistical analysis are mentioned in the graphs.

## Results

### Harsh touch response is affected by axotomy of PVD neuron but it remains unaffected by dendrotomy

PVD neuron displays an orthogonal array of dendritic branches that cover the major part of the body (Fig. 1*A*; Inberg et al., 2019). The higher-order branches are arranged in a menorah-like fashion (Fig. 1*A*; Oren-Suissa et al., 2010). Since both harsh touch sensation and proprioception are largely controlled by PVD neurons, it is not clear whether one or both modalities will be affected by dendrotomy or axotomy. Therefore, to understand whether dendrite regeneration would lead to functional restoration, it is important to have a quantitative behavioral deficit in a specific behavior caused due to dendrotomy. We first studied the effect of axonal and dendritic injury on the harsh touch response behavior. When the worms were prodded near the vulva with a platinum wire, they instantly exhibited an escape response (Fig. 1*B*, red mark, Movie 1) as seen before (Li et al., 2011). The distance traveled by the worm in each frame was shorter before the delivery of the harsh touch stimulus but it increased thereafter (Fig. 1*C*, Movie 1). Nearly 90% of the wild-type worms responded to the harsh touch (Fig. 1*D*) and the response remained comparable in the L4 and Day 1 stages, with a 20% decrease at the Day 2 stage (Fig. 1*D*). The percentage of animals responding to harsh touch was significantly reduced in *mec-3* mutant (Fig. 1*D*). The *mec-3* gene codes for a transcription factor, which is required for the expression of genes important for the development and function of PVD (Smith et al., 2013). However, this sensory behavior was unaffected in the gentle touch defective mutant of *mec-4* (Fig. 1*D*), which codes for the DEG/ENaC mechanoreceptor channel for gentle touch (Chalfie and Sulston, 1981). This indicated that our assay condition is specifically sensitive to harsh touch.

We ablated PVD neurons on one or both sides (PVDL and PVDR, Fig. 1*E,F*) at the L4 stage to assess the role of these neurons in harsh touch response. The percentage of worms responding to harsh touch was significantly reduced when both the PVD neurons (PVDL and PVDR) were ablated as compared to the control (mock injury) (Fig. 1*F*). However, in cases of ablation on one side, only the harsh touch given to ablated side resulted in a significant reduction in response (Fig. 1*F*). Similarly, we found that following axotomy near the cell body (red cross, Fig. 1*E,G*), the percentage of worms responding to harsh touch decreased when both the PVD neurons were axotomized and on the axotomized side when one axon was cut (Fig. 1*G*). Surprisingly, when we dendrotomized both the major and minor primary dendrites on both sides (PVDL and PVDR, Fig. 1*E,H*), the harsh touch response remained unaffected (Fig. 1*H*). This indicated that dendrotomy does not affect the harsh touch response behavior associated with PVD neurons.

We also developed a more quantitative assessment of harsh touch response by analyzing video recordings of the worm's response to a harsh touch stimulus. We used an Image-J plugin software (Nussbaum-Krammer et al., 2015) that tracks the worm's speed (Extended Data Fig. 1-1A). The speed of the locomotion increased significantly following the delivery of harsh touch (Extended Data Fig. 1-1B). The Day 1 and Day 2 adults also showed a similar increase in speed upon harsh touch (Extended Data Fig. 1-1B). We defined the harsh touch response index (HTRI) as the ratio of the worm's speed after to before the harsh touch delivery (Extended Data Fig. 1-1A). As expected, the HTRI value in the *mec-3* mutant is significantly lower than in the wild type (Extended Data Fig. 1-1C). On the other hand, the HTRI was unaffected in *mec-4* mutant worms compared to wild-type worms (Extended Data Fig. 1-1C). Additionally, HTRI levels were significantly lowered after PVD ablation or axotomy (Extended Data Fig. 1-1D,E). This decline was specific to the injured side (Extended Data Fig. 1-1D,E). Dendrotomy of major plus minor dendrites of both of the PVD neurons did not affect the HTRI value (Extended Data Fig. 1-1F). To answer the question that whether the dendritic morphology may play any role in harsh touch response or not, we checked the harsh touch response for the mutants that have a developmental defect in the formation of the dendritic arbor of PVD neuron such as *hpo-30(0)* and *tiam-1(0)* (Zou et al., 2018; Tang et al., 2019). The harsh touch response was unaffected in these mutants (Fig. 1*D*) saying that the dendritic morphology may not be playing any significant role in harsh touch response. This indicated that harsh touch sensory modality may not be entirely dependent on the dendrites of PVD neurons.

### The dendrotomy on PVD affects proprioception behavior

As the harsh touch response behavior was not affected by dendrotomy, we speculated that the proprioception behavior might be perturbed by dendrotomy. The dendrites release the neuropeptide NLP-12 to the neuromuscular junction that modulates the sinusoidal wave pattern formed during the movement of the worms on bacterial lawn (Tao et al., 2019). The amplitude and wavelength of the sinusoidal waveform made by a freely moving worm on the bacterial lawn (Fig. 2*A*) have been described as the hallmarks of proprioceptive behavior in worms (Albeg et al., 2011; Tao et al., 2019). These parameters are often altered in mutants missing higher-order dendritic branches in PVD neurons (Albeg et al., 2011; Tao et al., 2019). We used these parameters to test if this proprioception behavior was changed after dendrotomy. We took images of the sinusoidal traces made by the moving worms on the OP50 bacterial lawn on NGM plates (Fig. 2*A*). The amplitude, wavelength, and bending angle were computed from the sinusoidal trajectory (Fig. 2*A*). The *mec-3*, *hpo-30*, and *tiam-1* mutants, which have reduced number of higher-order branches in PVD (Zou et al., 2018; Sundararajan et al., 2019; Tang et al., 2019), exhibited a decrease in all postural measurements (Extended Data Fig. 2-1A–C). The mutant for *mec-10* which codes for DEG/ENaC channels also showed reduced values in these parameters (Extended Data Fig. 2-1A–C) as shown before (Tao et al., 2019). Ablation of the PVD cell body caused significant reduction in both the wavelength and amplitude of the waveform (Extended Data Fig. 2-1D–F). We found that the amplitude and wavelength values at Day 1 adult stage were significantly higher than those at larval stage L4 (Extended Data Fig. 2-1G–I), most likely due to an increase in the length of the worm body from 950 microns at L4 stage to 1.2 mm at Day 1 stage. These parameters were comparable in Day 1 and Day 2 stages (Extended Data Fig. 2-1G–I). Therefore, we decided to measure the changes in the parameters of body posture due to dendrotomy and regeneration at Day 1 and Day 2 stages, respectively.

We performed two types of dendrotomy experiments: (1) dendrotomy in major primary dendrites and (2) dendrotomy in both major and minor primary dendrites for both PVD (PVDL and PVDR, Fig. 2*B*). The trajectories of the worms that underwent dendrotomy appeared distinctly different as compared to the mock control (Fig. 2*C*). The postural parameters were decreased after dendrotomy of either major dendrites or major-plus-minor dendrotomy (Fig. 2*D,F*). The amplitude and bending angle were significantly decreased after the dendrotomy of the major primary (Fig. 2*D,F*). These parameters were reduced further significantly when both major and minor dendrites were cut, except the wavelength (Fig. 2*D,F*). The wavelength parameter was only perturbed with dendrotomy of major and minor dendrites (Fig. 2*E*). To see whether the defect in sinusoidal posture after laser injury is a secondary consequence of locomotion defect, we evaluated the baseline speed of the dendrotomized worms (Extended Data Fig. 2-1G). The speed of the worms remained unaltered postdendrotomy (Extended Data Fig. 2-1G). The gentle touch response was also unaffected in these worms (Extended Data Figure 2-1G, lower panel). When the PVD neurons were axotomized, we did not find any change in the postural parameters (Fig. 2*G–I*).

To ascertain if the dendrotomy-related drop in proprioception is nonspecific effect in one transgenic strain, we repeated this experiment in another transgenic strain expressing *mScarlet* diffusible reporter in PVD neurons (Extended Data Fig. 2-1H–L). The amplitude and bending angle both exhibited a reduction after dendrotomy of major as well as major-plus-minor dendrite in this strain (Extended Data Fig. 2-1I-L) whereas, the wavelength was dramatically reduced when both major and minor dendrites are severed (Extended Data Fig. 2-1K). We further assessed the relative contribution of PVDL and PVDR in the proprioception by injuring both major and minor dendrites on only one side (PVDL or PVDR) (Fig. 2*J*). When the injured PVD was in close-contact with the agar-surface of NGM plate, we observed a defect in the proprioception (Fig. 2*K–M*). Both the amplitude as well as wavelength parameters were affected in the one-side dendrotomy as compared to two-side dendrotomy experiments (Fig. 2*K–M*).

These experiments support the conclusion that the axons and dendrites of PVD neuron differentially regulate the harsh touch response and proprioception, respectively.

### Axon regrowth of PVD neurons restores the impairment in harsh touch response

**Figure 3.**
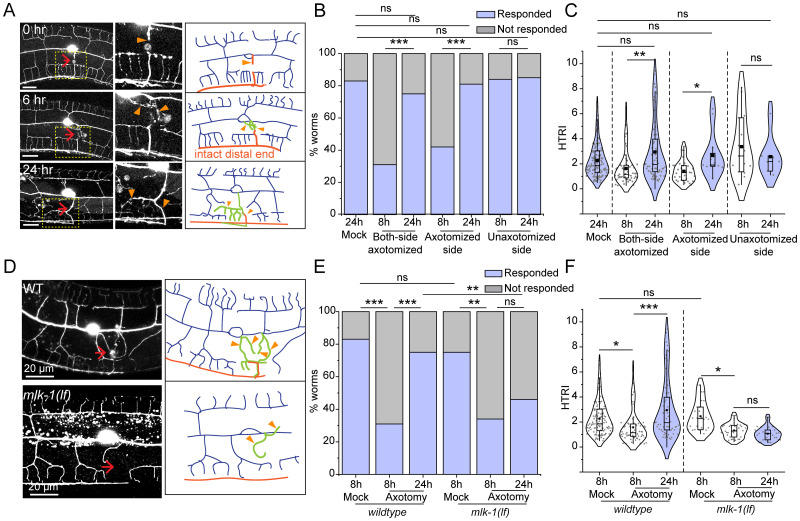
Axon regeneration leads to recovery in harsh touch defect. ***A***, Confocal images along with the schematics of PVD neuron is shown at 0, 6, and 24 h after axotomy representing the axon in orange, new protrusions in green traces, and orange arrowheads indicating the new regrowing branches. The red arrow marks the injury site. ***B,C***, Quantification of harsh touch as a percentage of worms responding (***B***) and harsh touch response indices (HTRI) (***C***) of mock (24 h), axotomized-side and nonaxotomy-side in one-side axotomy (8 and 24 h), and two-side axotomy (8 and 24 h) in *wdIs52 (pF49H12.4::GFP)* worms. 24 < *n* < 32, *N* = 3 (***B***). 21 < *n* < 35, *N* = 3 (***C***). The functional recovery at 24 h postsurgery was also followed up in case of the ablation of PVD (Extended Data Fig. 3-1). ***D***, The confocal images of regrowing axon in the wild-type and *mlk-1(0)* background at 24 h after injury. The red arrow marks the site of injury. ***E,F***, Worms responding to harsh touch as percentage (***E***) and harsh touch response indices (***F***) were quantified and plotted for mock at 8 h, axotomy at 8 h, and axotomy at 24 h after axotomy of both the PVD neurons in *wdIs52* and *mlk-1(0); wdIs52* background. 18 < *n* < 35, *N* = 3 (***E***). 18 < *n* < 35, *N* = 3 (***F***). The statistical analysis ***B***, and ***E***, is Fisher's exact two-tailed test, for ***C***, and ***F***, is one way ANOVA with Tukey's multiple comparison tests with *p* < 0.05*, 0.01**, and 0.001***. Violin plots represent the median and population distribution. ns stands for not significant, *N* stands for the number of independent replicates, and *n* stands for the number of worms taken for analysis.

Previous studies have elucidated that axon regeneration from the injured cut stump leads to functional recovery in various model systems (Becker et al., 1997; Laha et al., 2017; Rasmussen and Sagasti, 2017; Basu et al., 2021). As PVD neurons also show axonal regeneration (Brar et al., 2022), we investigated whether the deficits in the harsh touch response due to PVD axotomy would be restored in subsequent time points during the course of axon regeneration. After the axotomy near the cell body (red arrow, Fig. 3*A*), we saw a retraction of the injured tip, followed by new neurite formation and regrowth toward the ventral side (Fig. 3*A*). We noticed that in 67% cases, the distal part of the axons apparently looked intact (Fig. 3*A*, labeled in red color) and the regrowing neurites reconnected with the distal counterpart and fasciculated toward the ventral nerve cord (Fig. 3*A*). The proportion of worms responding to harsh touch at 24 h after axotomy was higher than at 8–10 h after axotomy in both one-side and two-side axotomy paradigms (Fig. 3*B*). Similarly, we estimated an increase in HTRI at 24 h postaxotomy in a side-specific manner (Fig. 3*C*). This rapid recovery in touch sensation could be partly mediated by the fusion between the proximal and distal dendrite as described in gentle touch neuron (Ghosh-Roy et al., 2010; Basu et al., 2017).

10.1523/ENEURO.0292-23.2024.f3-1 Fig 3-1 **Complete loss of harsh touch function due to ablation of PVD neurons.** Worms with one or both PVD ablated quantified as percentage responding to harsh touch (A) and harsh touch response indices (B) in conditions of harsh touch to mock (24  h), ablated-side and unablated-side in one-side ablation (8  h and 24  h) and, two-side ablation (8  h and 24  h) after injury. 24 < n < 32, N = 3 (A). 21 < n < 35, N = 3 (B). The statistical analysis for A is Fisher’s exact two-tailed test, and for B, is one-way ANOVA with Tukey’s multiple comparison tests with p < 0.05*, 0.01**, and 0.001***. Violin plots represent the median (red line) and population distribution. ns stands for not significant, N stands for the number of independent replicates, and n stands for the number of worms taken for analysis. Download Fig 3-1, TIF file.

DLK/MLK pathway is a critical regulator of axon regrowth following injury (Yan et al., 2009; Nix et al., 2011; Brar et al., 2022). To study if DLK/MLK mediated axonal regrowth is required for functional recovery, we assessed the harsh touch response in the *mlk-1* mutant (Fig. 3*D*). After axotomy, regrowth toward the ventral nerve cord was diminished in the *mlk-1* mutant (Fig. 3*D*). We noticed that the percentage of *mlk-1(0)* worms responding to harsh touch at 24 h did not increase as compared to 8 h after injury (Fig. 3*E,F*). The HTRI values indicated a recovery in harsh touch sensation at 24 h postinjury in the wild-type worms while this recovery was absent in the *mlk-1(0)* (Fig. 3*F*). This indicated a strong correlation between the axon regeneration to the recovery of harsh touch sensation in the PVD neurons. To ensure that the recovery in harsh touch response is indeed due to rewiring event, not due to some other phenomenon, we tested the recovery profile upon the complete removal of PVD in the wild-type worms (Extended Data Fig. 3-1A,B). When PVD was ablated, the harsh touch response did not improve at 24 h postablation as compared to response at 8 h postablation (Extended Data Fig. 3-1A,B). These results suggest that the axon regeneration in PVD neuron that is mediated by DLK/MLK pathway enables functional recovery of harsh touch sensation.

### Functional restoration during dendrite regeneration is correlated to successful events of arborization and fusion

**Figure 4.**
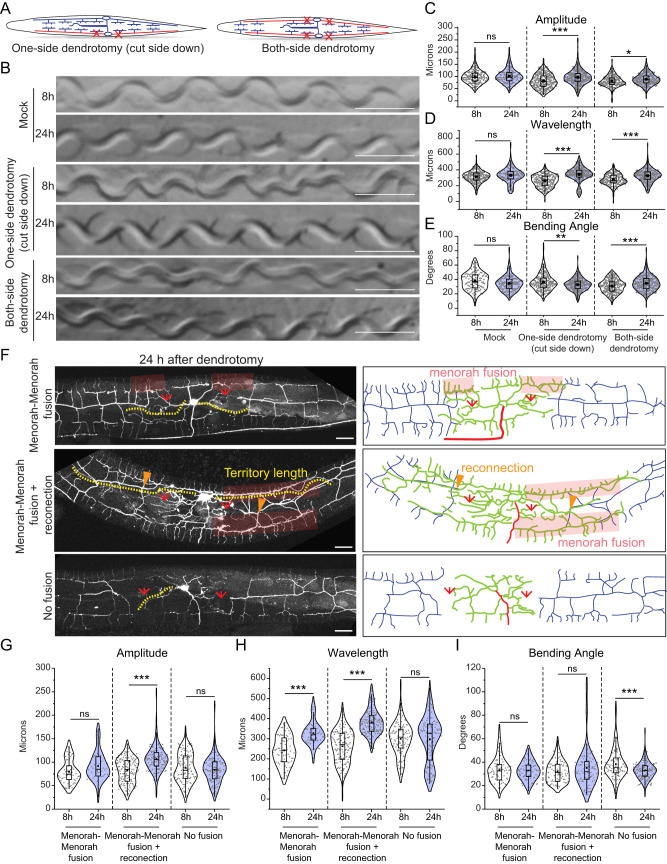
Dendrite regeneration in PVD neurons leads to recovery in posture defect of worms. ***A***, Schematics representing the one-side and both-side dendrotomy with both the major and minor dendrites were injured on one or both sides, respectively. ***B***, The images of the worm trajectories on the bacterial lawn are shown at 8 and 24 h after injury in mock, one-sided dendrotomy (cut-side down where the injured PVD is touching the agar media) and both-side dendrotomy conditions. The scale bar represents 500 microns. ***C–E***, Quantification of amplitude (***C***), wavelength (***D***), and bending angle (***E***) at 8 and 24 h after injury in mock, one-side dendrotomy (cut-side down, i.e., injured PVD neuron is touching the agar media) and two-side dendrotomy are plotted with the absolute values in a violin plots. The red dotted lines represent the median of population occurrence. 27 < *n* < 30, *N* = 3. The posture parameters were also correlated with the territory covered by regenerated dendrites and plotted in regression plot (Extended Data Fig. 4-1A–C). ***F***, Representative confocal images along with the schematics 24 h after dendrotomy with the menorah fusion highlighted by a red faded box, the green arrowheads marking the reconnection phenomena, and the red arrows representing the site of injury. The regenerated dendrites are represented in green, the distal part in grey, and the axon is represented in red in the schematic. The yellow dotted lines represent the longest regenerating dendrite corresponding to the territory covered by regenerated dendrites. Three different cases are shown, i.e., menorah fusion, menorah–menorah fusion plus reconnection, and no fusion events. ***G–I***, The quantification of postural parameters, i.e., amplitude (***G***), wavelength (***H***), and bending angle (***I***) are shown at 8 and 24 h after dendrotomy which are classified into three different cases which are menorah fusion, menorah fusion plus reconnection, and no fusion are plotted. 15 < *n* < 22, *N* = 3. The posture parameters were also quantified after the dendrotomy of wild type and *dlk-1(0);mlk-1(0)* worms (Extended Data Fig. 4-2A–D). The statistical analysis for ***C–E***, ***G–I*** is Tukey's multiple comparison tests, with *p* < 0.05*, 0.01**, and 0.001***. The violin plots represent the median and population distribution. ns stands for not significant, *N* stands for the number of independent replicates, and *n* stands for the number of worms taken for analysis.

Our previous study showed that the dendrites of PVD neurons can regenerate following a laser-assisted injury (Brar et al., 2022). However, it is unclear whether the relative change in the parameters involving proprioception would correlate with the events of successful dendrite regrowth. Dendrotomy of either one or both PVD neurons resulted in a drop in the amplitude and wavelength of the sinusoidal traces made by the moving worm (Fig. 4*A,B*). Qualitative inspection of the traces of dendrotomized worms showed a marked improvement at 24 h postdendrotomy with lesser irregularities in the sinusoidal movement as compared to the traces observed at 8 h postdendrotomy (Fig. 4*B*). All the parameters of proprioceptive behavior including amplitude, wavelength, and bending angle were significantly increased at 24 h postdendrotomy as compared to the values at 8 h (Fig. 4*C–E*).

10.1523/ENEURO.0292-23.2024.f4-1 Fig 4-1 **Characteristic pattern and extent of dendrite regeneration is correlated with recovery in postural parameters following injury** (A-C) The linear regression plot between various postural parameters such as amplitude (A), wavelength (B), and bending angle (C) to the territory length of regenerated dendrites. Regression analysis parameters are depicted in the plots with line of best fit, curved lines spanning the 95% confidence band and p values non zero regression fit. The cut-side down parameters were correlated with the territory length of one-side dendrotomy experiments. 10 < n < 32, N = 2. (D) The images of trajectories at 5-7hours and 24hours after dendrotomy at Day 1 *pser2prom3::mscarlet* worms in mock, dendrotomy in the major dendrite (both PVDs), and dendrotomy in the major plus minor dendrite (both PVDs) is shown. The scale bar represents 500 microns. (E) the confocal images along with schematics representing dendrite regeneration in green, the axon in red, and the distal part in grey color. The red arrow marks the site of injury, the faint red box represents menorah-menorah fusion and green arrowheads represent primary branch reconnection. The yellow dotted line represents the territory covered. The scale bar represents 10 microns. (F-H) Posture parameters i.e. amplitude(F), wavelength (G), and bending angle (H) are plotted in mock, dendrotomy in the major dendrite (both PVDs) and dendrotomy in the major plus minor dendrite (both PVDs) in Day 1 *pser2prom3::mscarlet* worms is shown, 15 < n < 20, N = 2. The absolute values were plotted with the median (red line) and population distribution. The statistical analysis for (A-C) is simple linear regression test with goodness of fit is calculated and its non-zero significance, (F-H) was one way ANOVA with Tukey’s multiple comparison test with p < 0.05*, 0.01**, and 0.001***. The violin plots represent the median and population distribution. ns stands for not significant, N stands for the number of independent replicates, and n stands for the number of worms taken for analysis. Download Fig 4-1, TIF file.

**Movie 1.** A freely moving worm of Day 1 stage on NGM plate was prodded with harsh touch pick. The Response of the worm was captured in this video at 15 frames/s and played at 30 frames/s.

Dendrite regeneration in the PVD neurons is accompanied by regrowth from the severed end, reconnection between distal and proximal counterparts, and fusion of the higher-order branches (Brar et al., 2022). The higher-order branches often fuse with each other to bypass the disjointed primary dendrites, which is termed as menorah–menorah fusion (Kravtsov et al., 2017; Oren-Suissa et al., 2017; Brar et al., 2022). In these experiments, we noticed a similar branching with reconnection (green arrowheads) and menorah–menorah fusion (red faded box) events (Fig. 4*F*). The regrowing dendrites span the gap created by the injury. This territorial expansion of the regrowing dendrites is estimated by the length of the longest neurite measured from the cell body (Yellow dotted line, Fig. 4*F*). We divided the events into three categories, “menorah–menorah fusion,” “menorah–menorah fusion plus reconnection,” and “no fusion/reconnection” (Fig. 4*F*). Based on the amplitude, wavelength, and bending angle measurements of the sinusoidal movement, significant increase in wavelength parameter was correlated to the “menorah fusion” events (Fig. 4*G–I*). In cases with “menorah fusion plus reconnection,” both amplitude and wavelength values were significantly increased at 24 h as compared to 8 h postdendrotomy (Fig. 4*G,H*). On the other hand, we did not observe any functional recovery in cases of “no fusion/reconnection” events (Fig. 4*G,H*). Interestingly, the bending angle did not change during dendrite regeneration except in cases of “no fusion/reconnection,” which showed a decrease (Fig. 4*I*). It is possible that loss of dendrites upon injury led to decline in the muscle or neuronal strength. This type of positive correlation between functional recovery and the “fusion” events is also observed after axonal injury in the gentle touch neurons (Abay et al., 2017; Basu et al., 2017). Additionally, there was a positive correlation between “territory length” and body posture parameters involving “amplitude” and “wavelength” (Extended Data Fig. 4-1A–C).

The proprioception parameters were also checked for both sides “major” and “major plus minor” dendrotomy experiments in worms with different transgene labeling PVD neurons with *mScarlet*. The trajectories on the OP50 lawn showed a visible recovery 24 h postinjury (Extended Data Fig. 4-1D). The regeneration events including reconnection and menorah fusion were also seen in these worms at 24 h postinjury (Extended Data Fig. 4-1E). We found that the affected parameters, i.e., amplitude and bending angle in case of major dendrotomy and all three parameters in case of major plus minor dendrotomy experiments showed an increase in value at 24 h after injury (Extended Data Fig. 4-1F–H).

Interestingly, the recovery in posture parameters was also observed in the double mutant for *dlk-1* and *mlk-1* (Extended Data Fig. 4-2). Overall, our analysis indicated that the worms can regain their posture that is lost due to dendrotomy through the regeneration of dendrites. This process can occur independent of DLK-/MLK-1 pathway.

10.1523/ENEURO.0292-23.2024.f4-2 Fig 4-2 **Recovery of posture parameters in absence of DLK/MLK pathway** (A) Confocal images along with the schematics that shows the regenerating dendrites in green, distal part in grey, the axon in orange and the site of injury with red arrows. The faint rectangular boxes represent the menorah-menorah fusion. (B) The proprioception parameters i.e. Amplitude, wavelength and Bending angle are measured and plotted in at 8  h mock, 8  h Both side Major and Minor dendrotomized worms, and 24  h Both side Major and Minor dendrotomized worms in wild type as well as *dlk-1(0);mlk-1(0)* double mutant. 15 < n < 23, N = 2. The statistical analysis for (A-C) is simple linear regression test with goodness of fit is calculated and its non-zero significance, (F-H) was one way ANOVA with Tukey’s multiple comparison test with p < 0.05*, 0.01**, and 0.001***. The violin plots represent the median and population distribution. ns stands for not significant, N stands for the number of independent replicates, and n stands for the number of worms taken for analysis. Download Fig 4-2, TIF file.

## Discussion

For faithful transduction of information in functional neuronal circuits, both dendrites and axons within an individual neuron play important roles (Blockus and Polleux, 2021). During the massive injury in the spinal cord, both axons and dendrites can get injured. In case of ischemia, stroke, and epilepsy, the dendritic arbors are prominently affected (Swann et al., 2000; Risher et al., 2010; Gao et al., 2011). The nerve graft-mediated stimulation of neurite regeneration in the injured spinal cord (Kumamaru et al., 2019) would also involve re-specification of the dendrites from the newly differentiated neurons ells in the graft. Therefore, functional rewiring after neuronal injury would require correct regrowth and integration of the regenerated dendrites to its presynaptic neuron or sensory organ. Although recent studies have shown that targeted injury on dendrites induces regrowth from the injured tip (Stone et al., 2014; Thompson-Peer et al., 2016; Brar et al., 2022), its functional consequence in adulthood is not clear. In this study, we systematically quantified the functional decline of both harsh touch response and proprioception following axon and dendrite injury on PVD neurons of *C. elegans*. Interestingly, we found that injury to the axon and dendrite affect harsh touch response and proprioception, respectively. This is consistent with the recent finding that dendrites and axons exclusively regulate two sensory modalities (Tao et al., 2019).

Moreover, we showed that axon regeneration leads to functional recovery in touch response in a DLK-1/MLK-1 pathway-dependent manner. On the other hand, dendrite regeneration leads to recovery in body posture defects caused due to dendrotomy and this process was independent of DLK-1/MLK-1 cascade. Previous studies also have indicated that the dendrite regeneration is independent of axon regeneration pathways such as DLK-1/MLK-1 (Stone et al., 2014; Brar et al., 2022), and it requires a unique mechanism involving GTPase and GEF activity (Brar et al., 2022). Dendrite regeneration in *Drosophila* da neurons is correlated to the functional recovery of nociceptive function in the larval stage (Hertzler et al., 2023). Moreover, recent studies in fish indicated a regeneration response after the injury to the dendrites of spinal motor neurons (Stone et al., 2022). In the mouse brachial plexus injury model, it is seen that dendrite regeneration is controlled by Rho GTPase (Li et al., 2023). Therefore, our findings in nematode highlight the functional importance of the dendrite regeneration process and its underlying molecular mechanisms.

We saw that the apparent fusion events between the proximal and distal dendrites highly correlated to the functional recovery in the body posture during locomotion. Similarly, functional recovery in harsh touch response postaxotomy could also be influenced by the fusion phenomena. Similar findings were seen in the case of repair of injured axons in earthworms, crayfish, leech, *Aplysia*, and nematode (Hoy et al., 1967; Birse and Bittner, 1976; Macagno et al., 1985; Bedi and Glanzman, 2001; Ghosh-Roy et al., 2010; Neumann et al., 2011). In *C. elegans*, the fusogen molecules such as EFF-1 and AFF-1 mediate the fusion process in axons and dendrites (Ghosh-Roy et al., 2010; Oren-Suissa et al., 2010; Neumann et al., 2015; Kravtsov et al., 2017). Here, we found that the recovery in some of the body posture parameters also correlated to the length of the territory covered by the regenerated dendrites.

Overall, our study using the PVD model establishes that dendrite regeneration can restore the lost sensory function due to injury in adulthood (see Visual Abstract). It further underscores the importance for studying a detailed molecular mechanism controlling the dendrite repair process.

## Synthesis

Reviewing Editor: Jeffery Twiss, University of South Carolina Decisions are customarily a result of the Reviewing Editor and the peer reviewers coming together and discussing their recommendations until a consensus is reached. When revisions are invited, a fact-based synthesis statement explaining their decision and outlining what is needed to prepare a revision will be listed below. The following reviewer(s) agreed to reveal their identity: NONE.

The field's understanding of dendrite regeneration is very preliminary. This manuscript describes a new assay to test for functional dendrite regeneration, in the PVD neurons of C. elegans. The authors contrast the function of PVD dendrites versus PVD axons, in terms of animal movement. They provide a compelling validation of a recent study (PMID: 31735664) that functionally separates distinct functional modalities to dendrites versus axons in the PVD neurons of C. elegans. These neurons have a stereotyped menorah-shaped dendritic arborization that covered the entire animal body length. These neurons have been shown to play important roles in both mechanical nociception, which requires its connection with downstream command neuron, and proprioception, which involves local neuropeptide release to modulate neuromuscular junction activity. The current study elegantly separates these two functions for these neurons using focal laser-induced injuries to the axon or dendrites of neurons. It is striking that removal of the axon abolishes a behavioral response to harsh touch without affecting proprioceptive-dependent sinusoidal movements, while dendrite removal has the complete opposite effect. Therefore one part of the neuron can still function in the complete absence of other part. This is a surprising yet compelling example of cellular compartmentalization of different circuit functions within a single cell. Similarly surprising is these behaviors show some recovery after 24 hours.

The reviewers and reviewing editor felt that this is a very nicely done story, with a simple and clear conclusion. Experiments are well-performed and well-described. In the initial reviewer critiques, one reviewers suggested additional experiments (outlined below) that you may want to consider performing. Alternatively, both the reviewers and reviewing editor voiced in the online discussion that providing some relevant speculation/discussion on these points would likely be sufficient.

Major points:

1. Figure 1D has the technical name of a GFP reporter (WdlS52) placed in the middle of the figure, which may lead readers from mammalian neural repair field to wonder whether the WldS transgene was present in the background for these experiments (reveiwers recognize this is C. elegans nomenclature for a transgenic line with the GFP expressed in the PVD neurons, but that was fully missed by the reviewing editor). The authors could instead define the reporter transgene in the methods and avoid possibility for misinterpretation by remvoing that label from the figure. It was also request that the authors ensure that the genetic details for all experiments are fully described in the materials and methods for the sake of rigor and reproducibility (also see 'Points to clarify' below).

2. The recovery of harsh touch response after axotomy is quite rapid, particularly considering that the axon has not regrown its full projection. Do the authors think that axon refusion is occuring? The authors should clarify this point either in the results or discussion as well as whether severed distal axons degenerate in C. elegans as in most other animals.

3. The author's penultimate sentence in the discussion, that their study 'establishes that axon and dendrite regeneration is both molecularly and functionally distinct', is not supported by the data. Their data do not directly demonstrate that axon and dendrite regeneration happen via different pathways. They show that axons and dendrites serve different purposes for these 2 behaviors (harsh touch versus proprioception), but they do not demonstrate that the injury detection or regeneration mechanisms are different. One reviewer suggested revising the conclusion to remove molecularly distinct description. They noted that there are other papers you can refer for this in the discussion, but the molecular mechanisms are not a focus of this current work and the distinction in the functions recovery for dendrite-versus axon regeneration is a sufficient point alone.

4. With regards to specific experiments that would strengthen the manuscript, the reviewers suggested: 1) PVD ablation and testing proprioception as in Fig 2, and 2) testing recovery of proprioception as i Fig 4 in mlk-1(0) mutants. These could provide more conclusive evidence to support your conclusions. As mentioned above, however, both the reviewers and reviewing editor leave this up to the authors whether to pursue these additional experiments or include some discussion on these possibilities.

5. Figure 5 could also serve as a visual abstract. It is informative and conveys the scientific message, but one reviewer described the flame-colored triangles as 'somewhat unusual'. The colors are not distinguishable on some printouts and screens (bright purple axons on the dendrite regeneration side is a bit distracting and light green regenerating axon is easy to miss). We recommend the authors change the colors, to better bring reader's attention to the regenerating neurites.

Points to clarify:

1. In the discussion, please more clearly articulate what is new about the findings in Fig 1 & Fig 2 from what was previously known. It seems like many of these findings of Fig 1 & Fig 2 were hinted at by previous work.

2. How long after dendrite injury is behavior tested? Is dendrite injury confirmed by imaging in **every** animal before behavioral response, or just for a few representative animals?

3. When assaying harsh touch and posture, are the same animals being tested?

4. Similarly, for the time course in Fig 3a, is this the same animal at 3 time points? The neurons look like they are different neurons at each time point.

5. Fig 1F: Is there a statistical difference between ablated side versus both side ablation? The ablated side response seems to indicate that lots of sensitivity comes from across the animals' body from the contralateral side.

6. The authors use the gentle touch assay in Supp Fig 2g, please include the gentle touch assay in the methods.

Minor points:

1. Typo: There are a couple of places where the authors refer to da9 neurons in Drosophila (line 76 and line 638). These are da neurons, not da9 neurons.

2. Does axotomy impair the gentle touch response?

3. Typo: Fig 3a, "schematics" is misspelled

4. Fig 5: specify that this is the "harsh" touch, to distinguish from gentle touch
