# Supplementary material for: Functional recovery associated with dendrite regeneration in PVD neuron of C. elegans
Source: eNeuro. Author manuscript; Available in PMC 2024 May 21. (PMC7615967; doi:10.1523/ENEURO.0292-23.2024)
Supplement: Supplementary file 1 [file EMS195576-supplement-Supplementary_information.pdf]

List of supplementary figures

Extended Data Figure 1-1: Harsh touch response index measured in different injury conditions of the PVD neurons

Extended Data Figure 2-1: The posture function of PVD neurons is correlated to its dendritic structure.

Extended Data Figure 3-1: Complete loss of harsh touch function due to ablation of PVD neurons.

Extended Data Figure 4-1: Characteristic pattern and extent of dendrite regeneration is correlated with recovery in postural parameters following injury

Extended Data Figure 4-2: Recovery of posture parameters in absence of DLK/MLK pathway

List of movies

Movie 1: Movie 1: Harsh touch assay in the worms.

List of Tables

Table 1 List of *C. elegans* strains used in this paper.

Table 2 List of strains carrying extrachromosomal transgenes used in this paper.

Extended Data Figure 1-1: Harsh touch response index measured in different injury conditions of the PVD neurons

(A) The formula of speed and Harsh touch response index (HTRI) is shown which was used in the analysis of injury experiments. The speed of the worm was measured considering 30 frames before and after harsh touch which was labeled as speed before harsh touch and speed after harsh touch, respectively. The ratio of these speeds was taken as HTRI. (B) The values of speed of uninjured worms are plotted before and after harsh touch at L4, Day 1, and Day 2 old stage worms on the NGM plates are shown in microns per second. $12<n<30$, N=3. (C) Harsh touch response indices are plotted for L4, Day1, Day2, L4 (*mec-3(0)*) and L4 (*mec-4(0)*) worms in *wdIs52 (pF49H12.4::GFP)* background. The violin plots represent the median (red line) and population distribution. $12<n<25$, N=3. (D-F) Harsh touch response indices are plotted for ablation (D), axotomy (E), and dendrotomy (F) experiments. Each violin plot in (D) represents mock, one side ablation (ablated-side, and unablated side), and both side ablation in *wdIs52 (pF49H12.4::GFP)* background. $15<n<25$, N=3. (E) plot represents mock, one side axotomy (axotomized-side, and non-axotomy side) and both side axotomy in *wdIs52 (pF49H12.4::GFP)* background. $13<n<35$, N=3 and (F) represents control (uncut), mock, and both side dendrotomy in *wdIs52 (pF49H12.4::GFP)* background. $12<n<28$, N=3. The violin plots represent the median (red line) and population distribution. The statistical analysis for (B-F), is one-way ANOVA with Tukey's multiple comparisons with p-value as $p < 0.05$*, 0.01**, and 0.001***. ns stands for not significant, N stands for the number of independent replicates, and n stands for the number of worms taken for behavioural study.

Extended Data Figure 2-1: The posture function of PVD neurons is correlated to its dendritic structure.

(A-C) Posture parameters of uninjured worms i.e. amplitude(A), wavelength (B), and bending angle (C) are plotted in *wdIs52, hpo-30(0);wdIs52, mec-3(0);wdIs52, mec-4(0);wdIs52, tiam-1(0) ;wdIs52 , and mec-10(0)* at L4 stage are plotted, $25<n<30$, N=3. The absolute values were plotted and the violin plots represent the median and population distribution. (D-F) Posture parameters of mock and PVD ablated worms i.e. amplitude(D), wavelength (E), and bending angle (F) are plotted in *wdIs52,* at L4 stage are plotted, $12<n<14$, N=3 . (G-I) Posture parameters such as amplitude (G), wavelength (H), and bending angle (I) are plotted in L4, Day1, and Day2 worms respectively. The absolute values were plotted and violin plots represent the median (red line) and population distribution. $15<n<25$, N>3. (J) The schematics showing the type of injury performed on *pserprom3::mscarlet* worms using a 2-Photon laser. The PVD neuron is labeled in red and the black cross represents the site of injury. (K-L) The baseline speed (K), as well as gentle touch response (L), were measured for the worms that

had undergone dendrotomy of major dendrite (both PVDs) and dendrotomy of major and minor dendrites (both PVDs) of *pser2prom3::mscarlet* worms, $13<n<25$, $N>3$. (M) The images of trajectories at 5-7h after dendrotomy at Day 1 stage *pser2prom3::mscarlet* worms in mock, Dendrotomy in the major dendrite (both PVDs) and dendrotomy in the major and minor dendrite (both PVDs) are shown. (N-P) Posture parameters i.e. amplitude (N), wavelength (O), and bending angle (P) are plotted in mock, dendrotomy in the major dendrite (both PVDs) and dendrotomy in the major and minor dendrite (both PVDs) in Day1 *pser2prom3::mscarlet* worms. $15<n<20$, N=2. The absolute values were plotted and the violin plots represent the median and population distribution. The statistical analysis for A-G, J-L is one-way ANOVA with Tukey’s multiple comparisons, and for (G-bottom panel) is –Fisher's two-tailed exact test with $p < 0.05$*, 0.01**, and 0.001***. The violin plots represent the population distribution. ns stands for not significant, N stands for the number of independent replicates, and n stands for the number of worms.

Extended Data Figure 3-1: Complete loss of harsh touch function due to ablation of PVD neurons.

Worms with one or both PVD ablated quantified as percentage responding to harsh touch (A) and harsh touch response indices (B) in conditions of harsh touch to mock (24h), ablated-side and unablated-side in one-side ablation (8h and 24h) and, two-side ablation (8h and 24h) after injury. $24<n<32$, N=3 (A). $21<n<35$, N=3 (B). The statistical analysis for A is Fisher’s exact two-tailed test, and for B, is one-way ANOVA with Tukey’s multiple comparison tests with $p<0.05$*, 0.01**, and 0.001***. Violin plots represent the median (red line) and population distribution. ns stands for not significant, N stands for the number of independent replicates, and n stands for the number of worms taken for analysis.

Extended Data Figure 4-1: Characteristic pattern and extent of dendrite regeneration is correlated with recovery in postural parameters following injury

(A-C) The linear regression plot between various postural parameters such as amplitude (A), wavelength (B), and bending angle (C) to the territory length of regenerated dendrites. Regression analysis parameters are depicted in the plots with line of best fit, curved lines spanning the 95% confidence band and p values non zero regression fit. The cut-side down parameters were correlated with the territory length of one-side dendrotomy experiments. $10<n<32$, N=2. (D) The images of trajectories at 5-7hours and 24hours after dendrotomy at Day 1 *pser2prom3::mscarlet* worms in mock, dendrotomy in the major dendrite (both PVDs), and dendrotomy in the major plus minor dendrite (both PVDs) is shown. The scale bar represents 500 microns. (E) the confocal images along with schematics representing dendrite regeneration in green, the axon in red, and the distal part in grey color. The red arrow marks

the site of injury, the faint red box represents menorah-menorah fusion and green arrowheads represent primary branch reconnection. The yellow dotted line represents the territory covered. The scale bar represents 10 microns. (F-H) Posture parameters i.e. amplitude(F), wavelength (G), and bending angle (H) are plotted in mock, dendrotomy in the major dendrite (both PVDs) and dendrotomy in the major plus minor dendrite (both PVDs) in Day 1 *pser2prom3::mscarlet* worms is shown , $15<n<20$, $N=2$. The absolute values were plotted with the median (red line) and population distribution. The statistical analysis for (A-C) is simple linear regression test with goodness of fit is calculated and its non-zero significance, (F-H) was one way ANOVA with Tukey's multiple comparison test with $p< 0.05$*, 0.01**, and 0.001***. The violin plots represent the median and population distribution. ns stands for not significant, N stands for the number of independent replicates, and n stands for the number of worms taken for analysis.

Extended Data Figure 4-2: Recovery of posture parameters in absence of DLK/MLK pathway

(A) Confocal images along with the schematics that shows the regenerating dendrites in green, distal part in grey, the axon in orange and the site of injury with red arrows. The faint rectangular boxes represent the menorah-menorah fusion. (B) The proprioception parameters i.e. Amplitude, wavelength and Bending angle are measured and plotted in at 8h mock, 8h Both side Major and Minor dendrotomized worms, and 24 h Both side Major and Minor dendrotomized worms in wild type as well as *dlk-1(0);mlk-1(0)* double mutant. $15<n<23$, $N=2$. The statistical analysis for (A-C) is simple linear regression test with goodness of fit is calculated and its non-zero significance, (F-H) was one way ANOVA with Tukey's multiple comparison test with $p< 0.05$*, 0.01**, and 0.001***. The violin plots represent the median and population distribution. ns stands for not significant, N stands for the number of independent replicates, and n stands for the number of worms taken for analysis.

Movie 1: Harsh touch assay in the worms.

Actively moving worms are touched in the middle using a platinum wire with cuboid tip. The impact point is marked with a red dot.

Table 1 List of C. elegans strains used in this paper

C. elegans **strains**	**Source**	**Identifier**
WdIs52(F49H12.4::GFP, unc119[+]) X	CGC	NC1687
pser2prom3::mscarlet; ttx3::RFP(ShrEx472)	NBRC	NBR95
mlk-1(ok2471)I	CGC	RB1908
tiam-1(ok772)I	CGC	RB907
hpo-30(ok2047)V	CGC	RB1657
mec-4(u253)X	CGC	TU253
mec-10(tm1552)X	CGC	ZB2551
mec-3(e1338)IV	CGC	CB1338

Table 2 List of strains carrying extra chromosomal array used in this paper

Strain	Transgene	DNA Construct	Genetic Background	DNA concentration
NBR95	*shrEx472*	pNBRGWY124 (*Pser2prom4[4.1kb]::mScarlet*)	*N2*	10ng/µl
